# Laser Thinning and Patterning of MoS_2_ with Layer-by-Layer Precision

**DOI:** 10.1038/s41598-017-15350-4

**Published:** 2017-11-14

**Authors:** Lili Hu, Xinyan Shan, Yanling Wu, Jimin Zhao, Xinghua Lu

**Affiliations:** 1 Institute of Physics, Chinese Academy of Sciences, Beijing, 100190 China; 2 Collaborative Innovation Center of Quantum Matter, Beijing, 100190 China; 3 School of Physical Sciences, University of Chinese Academy of Sciences, Beijing, 100049 China

## Abstract

The recently discovered novel properties of two dimensional materials largely rely on the layer-critical variation in their electronic structure and lattice symmetry. Achieving layer-by-layer precision patterning is thus crucial for junction fabrications and device engineering, which hitherto poses an unprecedented challenge. Here we demonstrate laser thinning and patterning with layer-by-layer precision in a two dimensional (2D) quantum material MoS_2_. Monolayer, bilayer and trilayer of MoS_2_ films are produced with precise vertical and lateral control, which removes the extruding barrier for fabricating novel three dimensional (3D) devices composed of diverse layers and patterns. By tuning the laser fluence and exposure time we demonstrate producing MoS_2_ patterns with designed layer numbers. The underlying physics mechanism is identified to be temperature-dependent evaporation of the MoS_2_ lattice, verified by our measurements and calculations. Our investigation paves way for 3D device fabrication based on 2D layered quantum materials.

## Introduction

Novel layered materials have attracted extensive interest since the discovery of graphene^1,2^ and the emergence of transition metal dichalcogenides (TMDs)^3–5^. The 2D materials quickly become a new platform for electronics^5–9^, optics^10–12^, optoelectronics^13,14^, magnetism^15^ and nanocatalysis^16–18^. Among them, MoS_2_ is considered to be one of the most typical and important TMDs, whose physical and chemical properties are widely explored. Atomically thin MoS_2_ films show high carrier mobility^9^, layer-dependent bandgap^3^, and various degrees of pseudospin^19^, based on which advanced optoelectronic devices can be developed. In tuning the electronic and optical properties of MoS_2_, layer number is one of the most important parameters. Thus obtaining MoS_2_ with designed layer numbers and patterns are pivotal for device fabrication and applications based on 2D materials.

However, the preparation of 2D materials with atomic layer precision and geometrical pattern is still a practical hinder and challenge so far. Methods with high efficiency, precise control, and reliable scalability are of pressing need. Currently, methods such as mechanical exfoliation^1^, liquid exfoliation^20,21^, chemical vapor deposition (CVD)^22^, and thermal etching^23^ have been used to produce 2D materials of atomic thickness. However they hardly simultaneously preserve both layer-number and in-plane pattern controllability. Laser ablation has relatively higher controllability and precision in fabricating nano-structures^24,25^. As an innovative and promising fabrication method, laser thinning together with patterning shows the potential of preparing atomically thin 2D films with lateral patterns. Graphene-related 2D materials are the most frequently investigated. By tuning the laser scanning time, multilayer graphene can be thinned layer-by-layer with femtosecond laser pulses^26,27^. Specifically, pioneering research showed that multilayer MoS_2_ can be fabricated down to a monolayer by laser thinning, with electronic properties comparable to the pristine monolayer^28^. Despite of the endeavors, due to the lack of general methodology for laser thinning of TMDs, so far preparation of MoS_2_ bilayer and trilayer by laser thinning has not been reported. Here we demonstrate unambiguously that monolayer, bilayer and trilayer of MoS_2_ can be fabricated by laser thinning and patterning. Furthermore, we uncover the thinning mechanism by both experimental investigations and theoretical calculations. We clearly show that layer and pattern thinning of MoS_2_, including bilayer and trilayer, is possible and practical by controlling the laser power and laser exposure time.

**Figure 1:**
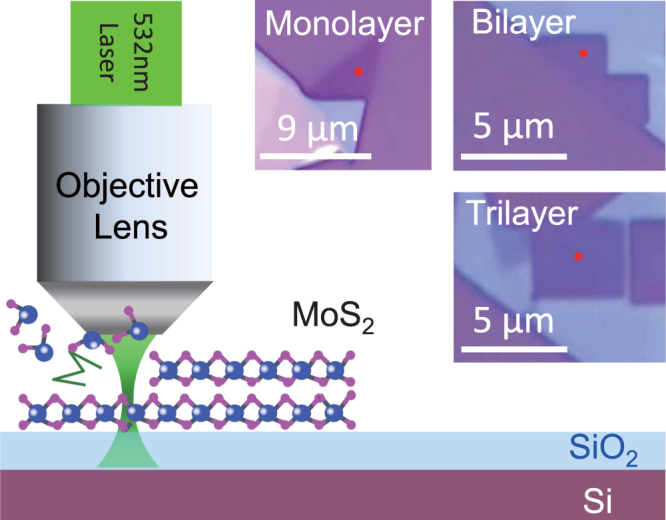
Schematic experimental setup of the laser thinning. Insets: optical images of laser thinning patterns composing monolayer, bilayer and trilayer of MoS_2_.

The schematic experimental setup and typical laser thinning pattern at different thicknesses of MoS_2_ are shown in Fig. 1. The experimental details are given in *Methods*. The thickness of the original films before thinning is chosen to be about 10 nm. Laser power and exposure time are the main parameters to control during the thinning and patterning. Raman spectra taken at a low laser power ( < 0.5 mW) are used, as a general means, to characterize the layer number of the MoS_2_ film. It is known that the frequency interval between the E_2*g*_ and A_1*g*_ peaks is 19.4 cm^−1^ for MoS_2_ monolayer, 21.2 cm^−1^ for bilayer, 23.2 cm^−1^ for trilayer and 25.3 cm^−1^ for bulk ones (*see* *Supporting Information* Fig. S1)^29,30^.

## Results

**Figure 2:**
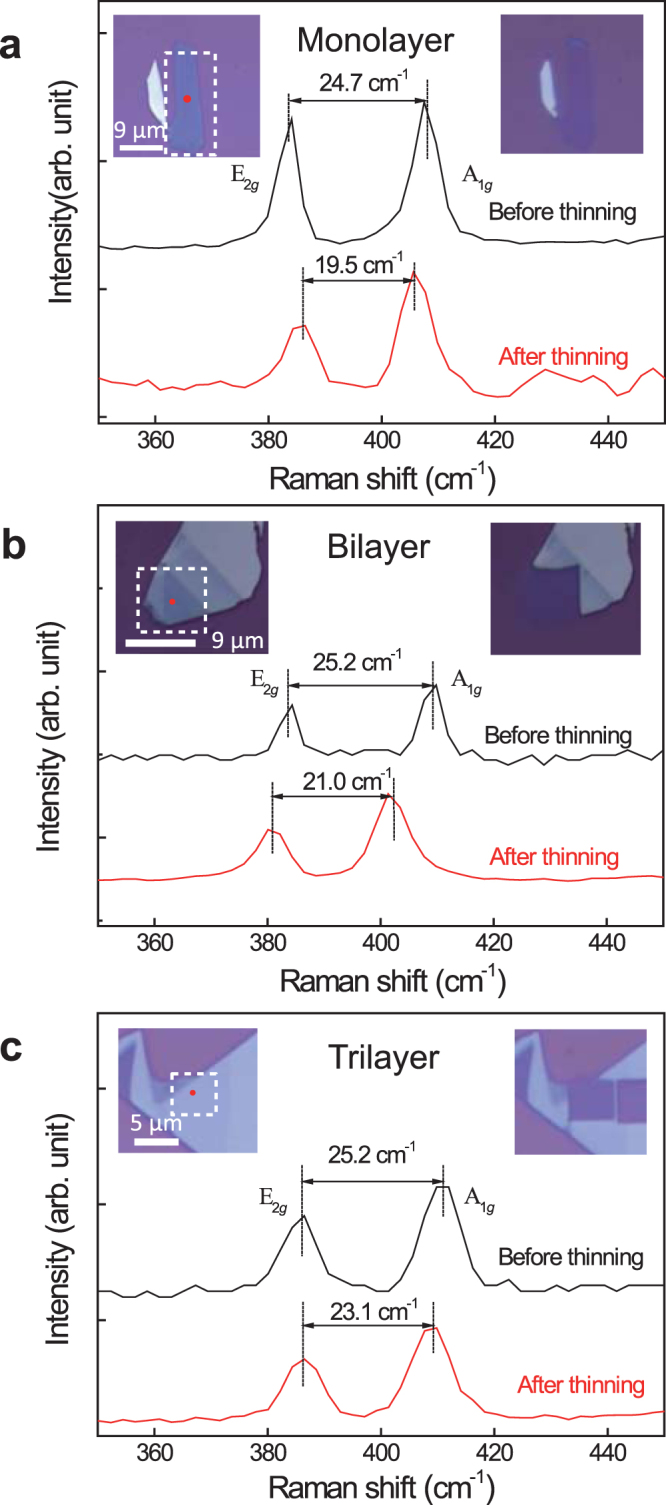
Achieving monolayer, bilayer, and trilayer MoS_2_ films. (**a**) Monolayer, with laser power of 2.5 mW and exposure time of 0.4 s at each pixel. (**b**) Bilayer, with laser power of 1.8 mW and exposure time of 0.4 s at each pixel. (**c**) Trilayer, with laser power of 2.5 mW and exposure time of 0.2 s at each pixel. The upper-left insets are optical images before laser thinning and the upper-right insets after laser thinning. The red dots denote the position where Raman spectra are collected. The Raman spectra are used to monitor the layer thickness.

In Fig. 2, we demonstrate that MoS_2_ films of monolayer, bilayer and trilayer can be fabricated. As shown in Fig. 2a, after 0.4 s at each pixel (0.2 µm × 0.2 µm) under the incidence laser power of 2.5 mW, the bulk film is thinned down to a monolayer film. The left and right insets are the optical images for the thin film before and after thinning, respectively. The laser scanning area is marked by a white rectangular dashed box. Before laser thinning, the frequency interval between the two Raman peaks (A_1*g*_ and E_2*g*_) is 24.7 cm^−1^, indicating a bulk nature of the film. After laser thinning, the frequency interval decreases to 19.5 cm^−1^, quantifying the thinned film to be a monolayer. In Fig. 2b, laser power of 1.8 mW is used to scan the white dashed box area with an exposure time 0.4 s at each pixel (0.2 µm × 0.2 µm). From the 21.0 cm^−1^ frequency interval between the A_1*g*_ and E_2*g*_ Raman peaks after the laser thinning, it can be identified that what left is a bilayer film. In Fig. 2c, laser power of 2.5 mW is used to thin the MoS_2_ bulk film down to a trilayer after an exposure time of 0.2 s at each pixel (0.2 µm × 0.2 µm), which is identified by observing the evolution of the Raman peak interval from 25.2 cm^−1^ into 23.1 cm^−1^.

**Figure 3:**
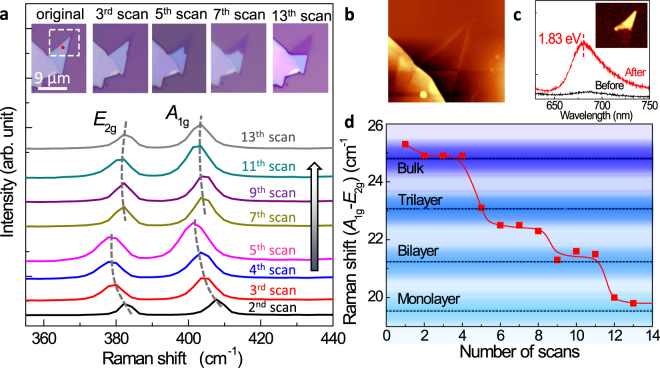
Controlling layer-by-layer thinning by tuning exposure time. (**a**) Evolution of layer number (monitored by Raman spectra) with increasing number of scans. For each scan, the laser power is 1.5 mW and the laser exposure time is 0.2 s. The insets show the patterns before and after the 3^rd^, 5^th^, 7^th^, and 13^th^ laser scan. (**b**) The AFM image after the 13^th^ scan. (**c**) The fluorescence spectra collected before and afer laser thinning. Inset: photoluminescence mapping image at the emission photon energy of 1.8 eV (690 nm) after the 13^th^ scan. (**d**) Dependence of the Raman peak interval (between the E_2*g*_ and A_1*g*_ modes, marking the layer thickness) on the number of laser scans.

Next we investigate laser thinning with layer-by-layer control. As shown in the insets of Fig. 3a, the scanning area is 10 µm × 10 µm separated into 50 × 50 pixels. The laser power used to do the well-controlled laser thinning is 1.5 mW, which is the lowest power we can observe the thinning effect. For each scan, the laser incidence lasts for 0.2 s at each pixel. As many as 13 scans are taken during the fabrication. Thus the total laser exposure time for each pixel is 2.6 s. During laser thinning, Raman spectra are taken simultaneously to monitor the number of layers, as shown in Fig. 3a. The atomic force microscope (AFM) topography (Fig. 3b) after laser thinning manifests atomically flat thickness. Our fluorescence measurement before and after laser thinning is shown in Fig. 3c. It can be seen that, after the thinning, there is a peak region with high efficiency fluorescence, which corresponds to the 1.8 eV direct band gap of monolayer MoS_2_^31^. In the inset of Fig. 3c, the fluorescence mapping is shown by recording the scattered photon centered at 1.8 eV. It also reveals the atomically flat thickness, as shown in Fig. 3b. At the given laser power of 1.5 mW, the layer number (and the corresponding frequency interval) depends on the number of scans, which is illustrated in Fig. 3d. After five scans, the bulk is thinned down to a trilayer; for six scans it is thinned down to a bilayer; and for 12 scans it is thinned down to a monolayer.

It can be seen from the optical images in the insets of Fig. 3a that laser thinning at 1.5 mW occurs initially at the edge of the bulk film, where the Mo and S atoms are less anchored and relatively easier to evaporate. Similar precursory edge evaporation has also been observed in the thermal etching of a bilayer MoS_2_ down to a monolayer at 330 °C^23^. Since the Raman spectra are taken at the spot marked by the red dot in the inset of Fig. 3a, the apparent shift between the 7–13^th^ scans and the 2–5^th^ scans is caused by the removal of the top layers of atoms. The reduced red-shift of Raman peaks (*i.e*. temperature) for the 7–13^th^ scans are likely due to higher heat dissipation into the substrate. It can be seen the largest red shift, compared to that without scanning, is 4.9 cm^−1^. Taking the reported relation $\frac{\partial \omega }{\partial T}=1.32\times {10}^{-2}c{m}^{-1}/K$ for multilayers^32^, we estimate the highest temperature due to the laser thinning to be 669 K (*see Supporting Information*), which is consistent with the reported minimum thermal thinning temperature of 603 K^23^. Taking the Raman peak value of 383.6 cm^−1^ after the 13^rd^ scans, the reported relation $\frac{\partial \omega }{\partial T}=1.1\times {10}^{-2}c{m}^{-1}/K$ ^32^ and ${\omega }_{297}$  = 384.3 cm^−1^ for a monolayer, we estimate the temperature of a monolayer heated by the laser beam (with an average power of 1.5 mW) to be 362 K. This temperature is much lower than the reported 603 K thermal thinning threshold, thus the thinned monolayer MoS_2_ is quite stable on substrate. Significantly, comparing with taking 15 hours to obtain a monolayer by thermal etching at 603 K^23^, it takes only seconds by laser thinning at room temperature in our experiment. This will greatly enhance the efficiency and feasibility of the fabrication for novel devices based on 2D quantum materials.

**Figure 4:**
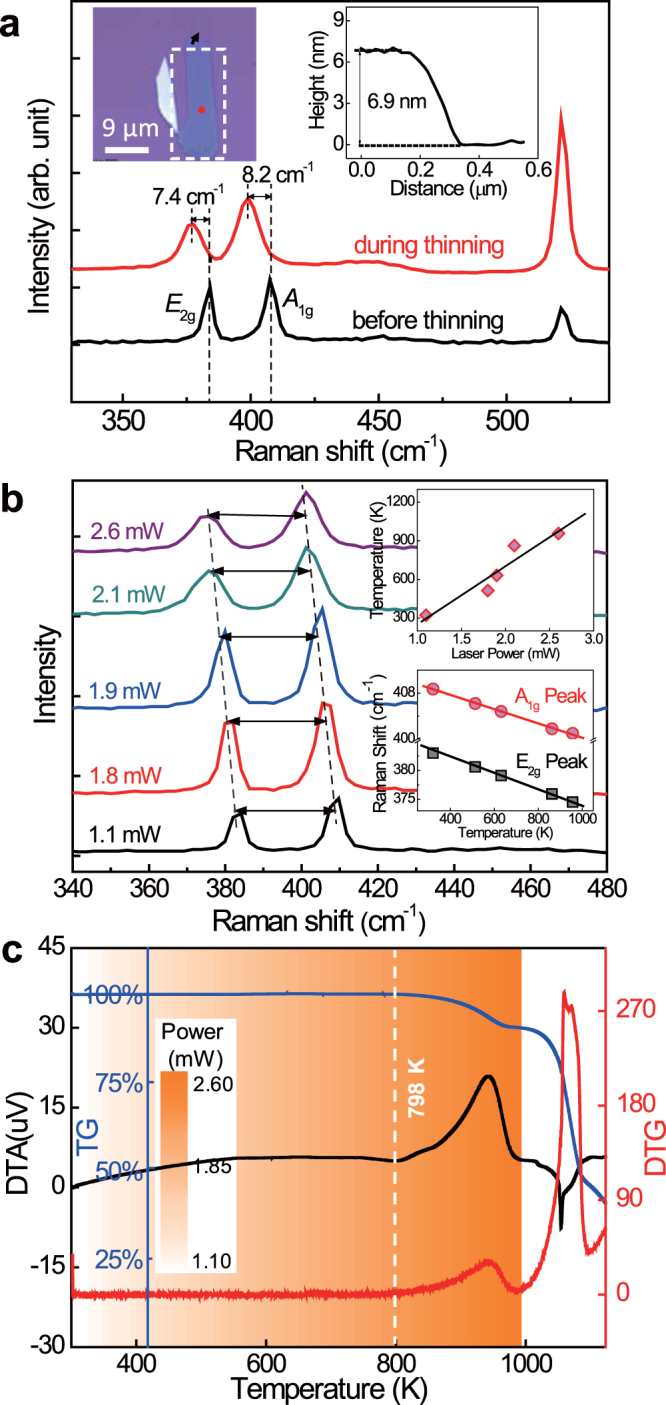
Physics mechanism of laser-induced thermal evaporation. (**a**) Red shift of Raman peaks revealing temperature enhancement. (**b**) Raman spectra under different laser powers. Upper inset: Proportional relation between temperature and laser power. Lower inset: Relation between estimated temperature and Raman shifts. (**c**) Evaporation rate at different temperatures, revealed by temperature-dependent weight-losing investigation. The black, red and blue curves are the DTA, TG and DTG curves, respectively. The temperature in (**b**) is also color-mapped onto the thermal gravimetric curves.

We then investigate the underlying mechanism of laser thinning through power-dependence and thermal gravimetric investigations. In Fig. 4a, the Raman spectra before and during the laser thinning are shown, where the laser power is 2.5 mW. The upper left inset shows the optical image of the sample area before laser thinning. We measured its height using AFM (upper right inset of Fig. 4a), which reveals a thickness of 11 layers. From the red shift of the E_2*g*_ peak (7.4 cm^−1^) we estimate that the temperature increases from room temperature to 859 K during the laser thinning (*see Supporting Information*). On the other hand, from the red shift of the A_1*g*_ peak (8.2 cm^−1^) we estimate that the temperature increases to 965 K (*see Supporting Information*). An average temperature of 912 K can be a more realistic estimation. The laser power-dependence of the temperature, where the temperature is read from the Raman spectra, is shown in Fig. 4b. In the upper inset of Fig. 4b we summarize the temperatures corresponding to different laser powers, which exhibit a linear relation. The relation between the Raman shift (for both E_2*g*_ and A_1*g*_ peaks) and estimated temperature is shown in the lower inset of Fig. 4b. Raman results for various temperature ranges have been reported in Refs.^33,34^ and many other papers, all exhibiting nearly identical relation. At last we also carry out the thermal gravimetric experiment, where we have air as the flowing gas. The results of differential thermal analysis (DTA), thermal gravimetric analysis (TG), and differential thermal gravimetric (DTG) are shown in Fig. 4c. We color-map the results in the inset of Fig. 4b into Fig. 4c, and compare it with the DTA, TG and DTG results. From the TG curve we know the weight loosing rate of MoS_2_ is sensitive to temperature variation at ~798 K (white dashed line), which corresponds to the laser power at 2.1 mW (see Fig. 4b). Thus Fig. 4c illustrates that the layer-precision thinning of MoS_2_ we have achieved (for example, see Fig. 3d) is indeed a result of laser-induced thermal evaporation of the MoS_2_ film. The reason we can achieve layer-by-layer control, including bilayer and trilayer, is that the weight loosing rate under laser heating depends on temperature.

## Discussion

We simulate the temperature distribution during laser thinning of a 10-layer MoS_2_ film. The three-dimensional temperature distribution is obtained by applying heat transfer equations to a MoS_2_ bulk film. Assume the thermal conductivity of air is negligible, the substrate has a constant room temperature, the film is a central symmetric disk with a radius *R = *2 μm and a layer number of *f * = 10, and the laser beam has a Gaussian profile. The temperature distribution *u*_*i*_ (t, r) of the *i*_*th*_ layer at time *t* and off center position *r* can be obtained using the following thermal diffusion equations

1 $${c}_{p}\cdot \rho \cdot \frac{d{u}_{1}}{dt}={I}_{1}+{k}_{r}\frac{{d}^{2}{u}_{1}}{d{r}^{2}}+\frac{{k}_{r}\frac{d{u}_{1}}{dr}}{r}-\frac{{k}_{z}({u}_{1}-{u}_{2})}{d{z}^{2}}$$

2 $${c}_{p}\cdot \rho \cdot \frac{d{u}_{i}}{dt}={I}_{i}+{k}_{r}\frac{{d}^{2}{u}_{i}}{d{r}^{2}}+\frac{{k}_{r}\frac{d{u}_{i}}{dr}}{r}-\frac{{k}_{z}(2{u}_{i}-{u}_{i-1}-{u}_{i+1})}{{dz}^{2}}$$

3 $${c}_{p}\cdot \rho \cdot \frac{d{u}_{f}}{dt}={I}_{f}+{k}_{r}\frac{{d}^{2}{u}_{f}}{d{r}^{2}}+\frac{{k}_{r}\frac{d{u}_{f}}{dr}}{r}-\frac{{k}_{z}({u}_{f}-{u}_{f-1})+{k}_{sub}({u}_{f}-RT)}{d{z}^{2}}$$

where *i* takes a value between 2 and *f-*1, *c*_*p*_ is the specific heat, *ρ* is the mass density and *I*_*i*_ is the light intensity that traverses the *i*_*th*_ layer. We have *I*_*i*_ = (1-*R*)·*I*_0_·*e* ^−*az*^. Taking temporal stepsize *dt* = 0.1 ns, spatial stepsize *dz* = 0.615 nm, which equals to half of the lattice distance, and the parameters in Table 1, we use a Mathematica package to calculate the temperature distribution.

**Table 1:** Parameters used for temperature distribution calculation.

Parameters	Value
Mass density of MoS_2_ *ρ*	5.1 × 10^−12^ g/μm^3^
Laser beam radius *r*_*g*_	0.13 μm
Reflectivity of MoS_2_ surface *R*	0.45
In-plane thermal conductivity *k*_*r*_	0.016 × 10^−3^ W/(μm·k)
Out-of-plane thermal conductivity *k*_*z*_	*k*_*r*_/100
Thermal conductivity between MoS_2_ and substrate *k*_*sub*_	0.4·*k*_*z*_
Gaussian beam with spatial distribution of intensity *I*_*i*_ *(r)*	$\frac{{I}_{{i}^{max}}\times {e}^{[-(2{r}^{2})/{r}_{g}^{2}]}\times 2}{\pi {r}_{g}^{2}}(i=1,2,\ldots f)$

**Figure 5:**
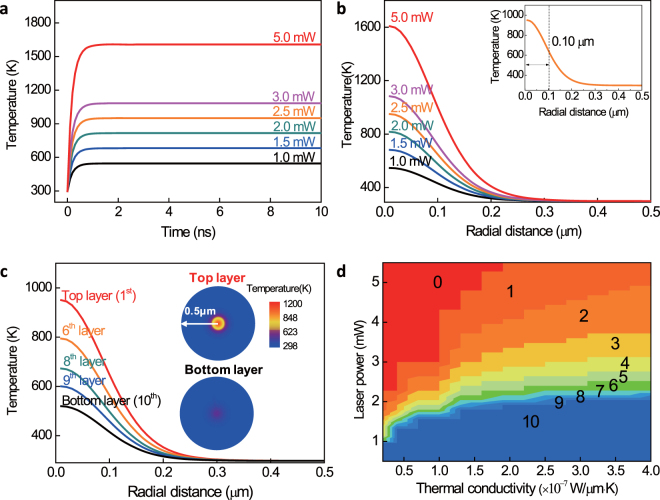
Simulation of the 3D temperature field evolution and distribution during laser thinning. (**a**) Temperature (of the top layer) evolution with time. (**b**) Spatial distribution of temperature (of the top layer). (**c**) Spatial distribution of temperature (of the top, 6^th^, 8^th^, 9^th^ and bottom layer). The laser power is 2.5 mW. Insets: temperature fields of the top and bottom layers. (**d**) Number of layer as a function of laser power and the thermal conductivity between MoS_2_ and substrate. We show the latter may be an additional engineering parameter in practical implementations.

The temperature evolution of MoS_2_ film during laser thinning is shown in Fig. 5a, which indicates that the temperature peaks and stabilizes in about 5 ns. We also obtain the in-plane spatial distribution of temperature for the top layer, as shown in Fig. 5b. The full width at half maximum  (FWHM) of temperature spatial distribution at 2.5 mW is 0.10 μm (inset of Fig. 5b), which is comparable to the laser beam radius 0.13 μm. As a result, our laser thinning can achieve very high spatial resolution, which is mainly limited by the laser beam foci size. The in-plane spatial distribution of temperature field for different stacked layers (along the *c*-axis) is shown in Fig. 5c, with laser power fixed at 2.5 mW. It can be seen that a prominent temperature difference, more than 100 K, exists between adjacent layers. Thus a precise layer-by-layer control is possible (see Fig. 4c). The in-plane spatial distribution of temperature for the top and bottom layers are shown in the insets of Fig. 5c. Since both laser heating and substrate cooling affects the temperature, another tuning parameter, the thermal conductivity between MoS_2_ and the substrates, can be included to improve the layer-by-layer precise thinning. As shown in Fig. 5d the number of layers below 798 K are plotted as a function of laser power and MoS_2_-substrate thermal conductivity. This is useful, in practical implementation of scalable laser thinning, in selecting substrate materials to accommodate the available/appropriate laser beam powers.

**Figure 6:**
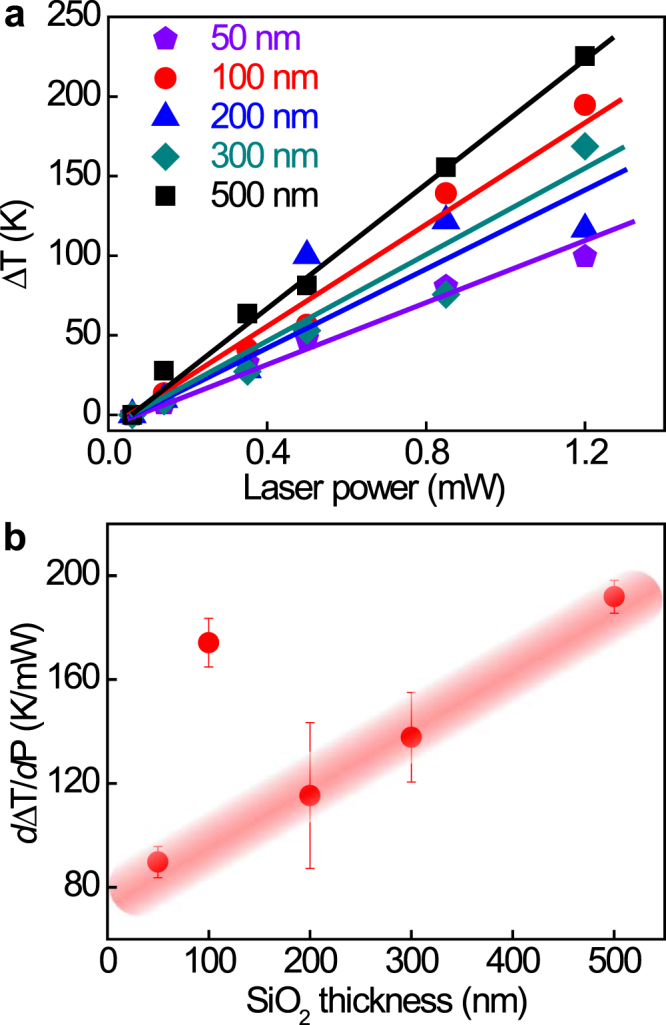
(**a**) Laser power dependence of temperature variation for a trilayer MoS_2_ film on Si/SiO_2_ substrates of different thicknesses of SiO_2_ layer. The temperature increase is obtained from the Raman shifts of the E_2*g*_ and A_1*g*_ modes. (**b**) Slopes in Fig. 6a as a function of SiO_2_ thickness.

Since substrates with different SiO_2_ thicknesses are often used in MoS_2_ investigation, we finally investigate the influence of SiO_2_ thickness on laser thinning and patterning. We show the laser power dependence of temperature increase for a MoS_2_ trilayer on different substrates (*i.e.* SiO_2_/Si substrates with different SiO_2_ capping layer thicknesses *d*_SiO2_ = 50 nm, 100 nm, 200 nm, 300 nm, 500 nm), respectively, in Fig. 6a. Again, the temperature is obtained from the Raman shift of both the E_2*g*_ and A_1*g*_ peaks. Other than the linear temperature-dependence observed in Fig. 4b, we observe different slopes for different substrates. To see this clearly, we explicitly plot the slops as a function of *d*_SiO2_, which is shown in Fig. 6b. Definite positive correlation (close to linear) is observed between *d*ΔT/*d*P and *d*_SiO2_, where P is the laser power. This can be explained by that the SiO_2_ layer acts as a thermal insulator between the MoS_2_ film and the Si heat sink. Note that the slope *d*ΔT/*d*P is the heating efficiency (*i.e*. temperature increase per unit incident laser power). Interestingly, an extraordinarily large slope is observed for the *d*_SiO2_ = 100 nm case (Fig. 6). This is indeed a more efficient laser heating phenomena, which we attribute to the enhanced laser reflection due to the Fresnel interference. Here the SiO_2_ layer affects the cooling and light absorption of the thin MoS_2_ film. Thus in practice the thickness of the SiO_2_ layer can be chosen as a means to control the efficiency of the laser thinning and patterning.

In this paper, we demonstrate that MoS_2_ bulk films can be thinned down to monolayer, bilayer and trilayer, and other designed numbers of layers by simply tuning the laser power and exposure time. We demonstrate lateral patterning of MoS_2_ thin films can be achieved with a precision of less than 0.26 μm. For the first time we are able to quantitatively design (by tuning the laser power and exposure time) such layer-by-layer precision thinning and patterning, with unprecedented short fabrication time compared with other methods, which together enable laser thinning an ideal method to fabricate nano-devices based on layered quantum materials. We also found the underlying physics mechanism is laser-induced thermal evaporation. Our investigation removes current obstacles for efficient fabrication of 3D TMDs-based nano-devices, thus paving way for applications of quantum materials in various cutting-edge areas.

## Methods

High quality MoS_2_ flakes are mechanically exfoliated from a MoS_2_ single crystal and transferred onto a SiO_2_/Si substrate (the thickness of the SiO_2_ layer is 285 nm except for substrate variation experiment). Laser thinning experiments are carried out using a confocal Raman spectrometer (WITec, alpha 300) in ambient environment. As shown in Fig. 1, continuous wave laser beam at 532 nm is focused by a 100×objective lens (Zeiss, NA = 0.9) onto a MoS_2_ bulk film, with a focal diameter of 260 nm. Mapping mode of the Raman spectrometer is used for laser patterning. A 1200 *l/mm* grating is used for Raman spectra while a 600 *l/mm* grating is used for taking fluorescence spectra. The AFM topography before and after laser thinning is taken in the ac mode.

## Electronic supplementary material

Supplementary Information
