# Supplementary material for: Laser Thinning and Patterning of MoS2 with Layer-by-Layer Precision
Source: Sci Rep. 2017 Nov 14;7:15538. doi: 10.1038/s41598-017-15350-4 (PMC5686209; doi:10.1038/s41598-017-15350-4)
Supplement: Supplementary file 1 — Supplementary Information [file 41598_2017_15350_MOESM1_ESM.doc]

Supporting Information for:

**Laser Thinning and Patterning of MoS_2_ with Layer-by-Layer Precision**

Lili Hu^1^, Xinyan Shan^1,3^, Yanling Wu^1^, Jimin Zhao^1,3^*, Xinghua Lu^1,2,3^*

*^1^ Institute of Physics, Chinese Academy of Sciences, Beijing 100190, China*

*^2^Collaborative Innovation Center of Quantum Matter, Beijing 100190, China*

*^3^School of Physical Sciences, University of Chinese Academy of Sciences, Beijing 100049, China*

Corresponding authors: [jmzhao@iphy.ac.cn](mailto:jmzhao@iphy.ac.cn), [xhlu@iphy.ac.cn](mailto:xhlu@iphy.ac.cn)

1. **Raman spectra of pristine monolayer, bilayer, trilayer and bulk MoS_2_**

The identification of the layer number is achieved by measuring the Raman peak interval between the E_2_*_g_* and A_1_*_g_* modes. Here we collect the Raman spectra of pristine monolayer, bilayer, trilayer and bulk MoS_2_ freshly exfoliated from a MoS_2_ single crystal. The results are illustrated in Figure S1.

Figure S1. Raman spectra for pristine monolayer, bilayer, trilayer and bulk MoS_2_ film, which are freshly exfoliated from a MoS_2_ single crystal.

1. **Temperature estimation from Raman spectra**

The temperature estimation in the main text is based on the following equation

$\omega(T)=\omega_{T_{0}}+\chi\cdot(T-T_{0})$ ,

where $\omega(T)$ is the peak value of E_2_*_g_* (or A_1_*_g_*) at temperature *T*, $\omega_{T_{0}}$is the peak position value at temperature *T*_0_, and *χ* is the coefficient of frequency change against temperature variation. The reported *χ* values are listed in Table S1.

Table S1: Coefficient of frequency change against temperature variation (*χ*) from references.

| Coefficient | Mode | few layer^s1^ | bulk^s2^ | monolayer^s3-s4^ |
| --- | --- | --- | --- | --- |
| *χ* (×10^-2^ cm^-1^/K) | E_2_*_g_* | -1.32 | -1.47 | -1.1^s3^  -0.97^s4^ |
|  | A_1_*_g_* | -1.23 | -1.23 | -1.3^s3^ |

We measure the Raman shift of the E_2_*_g_* mode to be *ω*_298k_ = 383.5 cm^-1^ for the first scan, and *ω*_298k_ = 384.3 cm^-1^ for monolayer at room temperature. With these Raman values we measured and the coefficients (Table S1) reported, we are able to obtain the estimated temperatures after different number of scans as shown in Figure 3. We assume the *χ* value persists for higher temperatures up to 1000 K and summarize the temperatures in Table S2.

Table S2: Temperatures estimated from the Raman spectra in Figure 3.

| Value of E_2_*_g_* Raman peaks (cm^-1^) | Temperature estimated from E_2_*_g_* peaks (K) |
| --- | --- |
| 383.5 (1^st^ scan)  383.2 (2^nd^ scan)  379.3 (3^rd^ scan)  378.6 (4^th^ scan)  378.6 (5^th^ scan)  379.1 (6^th^ scan)  381.7 (7^th^ scan)  381.0 (8^th^ scan)  382.0 (9^th^ scan)  380.4 (10^th^ scan)  381.1 (11^th^ scan)  382.7 (12^th^ scan)  383.6 (13^th^ scan) | 298  321  616  669  669  631  434  487  411  533  480  392  362 |

We then estimate the temperature for the results shown in Figure 4a. We have two Raman peaks that can be used to estimate the temperature. As shown in Table S3, from the E_2_*_g_* and A_1_*_g_* peaks, we estimate the temperature to be 859 K and 965 K, respectively. As a rough estimation, we take the average of the two, which is 912 K.

Table S3: Temperature estimation for the results shown in Figure 4a.

|  | Red shift (cm^-1^) | *χ* (×10^-2^ cm^-1^/K) | *T* (K) |
| --- | --- | --- | --- |
| E_2_*_g_* | 7.4 | -1.32 | 859 |
| A_1_*_g_* | 8.2 | -1.23 | 965 |

Similarly we estimate the temperatures for results shown in Figure 4b, which are shown in Table S4.

Table S4: Temperature estimation for the results shown in Figure 4b.

| Laser power  (mW) | E_2_*_g_* Raman shift  (cm^-1^) | A_1_*_g_* Raman shift  (cm^-1^) | Temperature estimated (K) |
| --- | --- | --- | --- |
| 1.1  1.8  1.9  2.1  2.6 | 383.0  380.6  379.1  375.9  374.5 | 408.7  406.2  404.8  401.8  401.0 | 321  513  631  862  956 |

**References**

s1. Sahoo, S.; Gaur, A. S. P.; Ahmadi, M.; Guinel, M. J. -F.; Katiyar, R. S. *J. Phys. Chem. C.* **2013**, 117(17), 9042-9047.

s2. Wilson, J. A.; Yoffe, A. D. *Advances in Physics* **1969**,18(73),193-335.

s3. Yan, R.; Simpson, J. R.; Bertolazzi, S.; Brivio, J.; Watson, M.; Wu, X. F.; Kis, A.; Luo, T. F.; Walker, A. R. H.; Xing, H. G. *ACS Nano* **2014**, 8(1), 986-993.

s4. Taube, A.; Judek, J.; Lapinska, A.; Zdrojek, M.; *ACS Appl. Mater. Interfaces.* **2015**, 7(9), 5061-5065.
